# Geno- and phenotypic characteristics and clinical outcomes of *CACNA1C* gene mutation associated Timothy syndrome, “cardiac only” Timothy syndrome and isolated long QT syndrome 8: A systematic review

**DOI:** 10.3389/fcvm.2022.1021009

**Published:** 2022-11-29

**Authors:** János Borbás, Máté Vámos, Lidia Hategan, Lilla Hanák, Nelli Farkas, Zsolt Szakács, Dezső Csupor, Bálint Tél, Péter Kupó, Beáta Csányi, Viktória Nagy, András Komócsi, Tamás Habon, Péter Hegyi, Róbert Sepp

**Affiliations:** ^1^Division of Non-Invasive Cardiology, Department of Internal Medicine, University of Szeged, Member of the European Reference Network for Rare, Low Prevalence, or Complex Diseases of the Heart (ERN GUARD Heart), Szeged, Hungary; ^2^Institute for Translational Medicine, Medical School, University of Pécs, Pécs, Hungary; ^3^Institute of Bioanalysis, Medical School, University of Pécs, Pécs, Hungary; ^4^Division of Cardiology, First Department of Medicine, Medical School, University of Pécs, Pécs, Hungary; ^5^Institute of Clinical Pharmacy, University of Szeged, Szeged, Hungary; ^6^1st Department of Pediatrics, Semmelweis University, Budapest, Hungary; ^7^Heart Institute, Medical School, University of Pécs, Pécs, Hungary; ^8^Szentágothai Research Centre, University of Pécs, Pécs, Hungary; ^9^Centre for Translational Medicine, Semmelweis University, Budapest, Hungary; ^10^Division of Translational Medicine, Department of Internal Medicine, University of Szeged, Szeged, Hungary

**Keywords:** *CACNA1C* gene, Timothy syndrome, mortality, long QT syndrome, L-type calcium channel, mutation

## Abstract

**Background:**

Mutations in the *CACNA1C* gene–encoding for the major Ca^2+^ channel of the heart–may exhibit a variety of clinical manifestations. These include typical or atypical Timothy syndromes (TS) which are associated with multiple organ manifestations, and cardiac involvement in form of malignant arrhythmias, QTc prolongation, or AV block. “Cardiac only” Timothy syndrome (COTS) shows no extracardiac manifestation, whereas some *CACNA1C* gene mutations are associated with QTc prolongation alone (isolated long QT syndrome 8, LQT8).

**Methods:**

A systematic search of the literature reporting cases of *CACNA1C* gene mutation associated syndromes, including TS, COTS and isolated LQT8 *via* major databases published from 2004 through 2019 was performed. Detailed patient-level phenotypic and genotypic characteristics, as well as long-term outcome measures were collected and compared between pre-specified patient groups, defined both on phenotype and genotype.

**Results:**

A total of 59 TS, 6 COTS, and 20 isolated LQT8 index cases were identified. Apart of syndactyly or baldness, there were no major differences regarding clinical manifestations or outcome measures between TS subtypes, either defining TS subtypes on the genotype or based on the phenotype. Both subtypes were characterized by an extreme degree of QTc prolongation (median ≥600 ms) which were reflected in high major adverse cardiac event rate. On the other hand, there were marked differences between TS, COTS, and isolated LQT8. Timothy syndrome was characterized by a much earlier disease onset, much more pronounced QTc prolongation and much higher mortality rate than COTS or isolated LQT8. Similar differences were observed comparing *CACNA1C* exon 8/8A vs. non-exon 8/8A mutation carriers. TS showed a high degree of genetic homogeneity, as the p.Gly406Arg mutation either in exon 8 or exon 8A alone was responsible for 70% of the cases.

**Conclusions:**

Clinical phenotypes associated with mutations in the *CACNA1C* gene show important clinical differences. Timothy syndrome is associated with the most severe clinical phenotype and with the highest risk of morbidity and mortality. However, distinguishing TS subtypes, in any form, are not supported by our data.

**Systematic review registration:**

[https://www.crd.york.ac.uk/prospero/], identifier [CRD42020184737].

## Introduction

In cardiac myocytes, inwardly rectifying Ca^2+^ current passes through the L-type calcium channel, the Ca_*v*_1.2 voltage-gated calcium channel, thereby initiating calcium release through activation of ryanodine receptor 2 (RyR2) from the sarcoplasmic reticulum. The channel has several subunits, including the α1C-subunit, responsible for voltage sensing, for conduction pore formation, and for the gating mechanism. The fully functional channel complex is comprised of an additional intracellular β subunit and an extracellular α2δ subunit. The α1C subunit has 4 domains (I–IV), each with six transmembrane-spanning segments (S1–S6). S1–S4 are the voltage-sensitive subunits, whereas S5–S6 are the selectivity filter. When the membrane is depolarized, the S4 domain approaches the cytoplasmic ends of the S5 and S6 helices and the conduction pore opens. Changes in L-type calcium channel function (*via* phosphorylation, mutations, drugs, etc.) can affect cardiac myocyte contractility and arrhythmogenicity. The channel is expressed in many tissues of the body (1–3).

The α1C subunit of the channel is encoded by the *CACNA1C* gene. Mutations affecting the *CACNA1C* gene may exhibit a variety of clinical manifestations. These manifestations include the typical Timothy syndrome (Timothy syndrome 1, TS1), which is characterized by QTc prolongation, AV-block, congenital heart defects, facial dysmorphisms, episodic hypoglycemia and neurologic symptoms including developmental delays, possible autism, seizures, and intellectual disability. The most distinctive morphological hallmark of TS1 is syndactyly, in contrast to atypical Timothy syndrome (Timothy syndrome 2, TS2) patients, who have no syndactyly but carry many of the other multisystemic manifestations of the disease [recently reviewed by Bauer et al. (4)]. Further clinical manifestation of *CACNA1C* gene mutations include “cardiac only” Timothy syndrome (COTS) (5), which is characterized by QTc prolongation and congenital heart defects without extra-cardiac manifestations. In contrast to the above mentioned phenotypes, some *CACNA1C* gene mutations are associated with isolated QTc prolongation (isolated long QT syndrome 8, LQT8), exhibiting QTc prolongation only without additional cardiac or extra-cardiac manifestations (6–8). Because the *CACNA1C* gene was the eighth gene proved to cause QTc prolongation, it was historically called LQT8, but today a clear distinction exists between multi-organ Timothy syndrome and isolated LQT8 (9).

**FIGURE 1:**
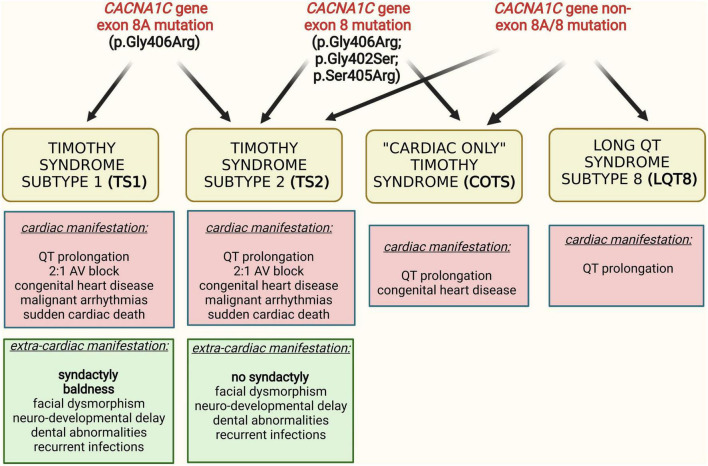
Interrelation of different clinical phenotypes and genotypes in *CACNA1C* gene mutation associated diseases.

These phenotypes are associated with a variety of *CACNA1C* gene mutations, affecting different regions of the *CACNA1C* gene. The predominant genetic cause of TS1, identified in 2004, is a recurrent, canonical “*de novo*” heterozygous missense mutation, p.Gly406Arg, in the alternatively spliced exon 8A (10). In cases with TS2, additional mutations, originally p.Gly406Arg and p.Gly402Ser, were identified in exon 8 of the gene (11). With the clinical description of “cardiac only” TS and isolated LQT8, an increasing number of *CACNA1C* mutations have been described in the literature. The accumulated data indicated a great amount of genetic and phenotypic heterogeneity of *CACNA1C* mutations associated phenotypes, i.e., “typical” TS mutations showing variant phenotypes (12) and “non-typical” TS mutations associated with typical TS phenotype (13–16) (Figure 1). Consequently, there is a high degree of controversy regarding the classification of *CACNA1C* gene associated phenotypes, especially regarding TS, mainly due to the lack of comparable outcome data (4).

Since TS is an extremely rare disease and most of the respective publications comprised only individual cases or case series, a systematic review of published data appears to be the best way to summarize the typical characteristics of this genetic disorder and to describe any possible correlations between the different phenotypes and clinical outcomes. Accordingly, the aim of the current work was to identify correlations between the mutations in different regions of the *CACNA1C* gene and various morphologic manifestations with different clinical outcomes. A special attention was made to the subject as to whether clinically meaningful differences exist among the different subtypes of Timothy syndrome.

## Methods

This systematic review was reported in accordance with the PRISMA Statement for reporting systematic reviews and meta-analyses (17). Our predefined review protocol was published in the PROSPERO database under the registration number of CRD42020184737. There was no deviation from the predefined and published protocol during the study.

### Systematic search

A comprehensive search was conducted in MEDLINE (*via* PubMed), Embase, Web of Science, and Scopus databases from 2004 through 2019 focusing on full-text papers published reporting data on patients with Timothy syndrome or isolated long QT syndrome 8 (LQT8) affected by mutations of the *CACNA1C* gene. Conference abstracts were included when same data could be extracted as from full-text papers. Studies eligible for inclusion were identified by using the following search query as full text search: “Timothy syndrome” OR (”LQT8 OR *CACNA1C*”).

### Eligibility criteria and selection

The eligibility criteria for this systematic review were as follows:

1) Reporting data on patients and/or relatives with documented mutation of the *CACNA1C* gene in the English language;
2) Describing detailed geno- and/or phenotypic features of the case;
3) Reporting data on clinical outcomes.

Reports on *CACNA1C* gene mutation associated Brugada syndrome (BrS3), short QT syndrome (SQT4) or early repolarization syndromes (ERS), exhibiting fundamentally different pathomechanisms, were excluded. Duplicate cases were identified, and the less informative ones were excluded. Two review authors independently evaluated all potentially relevant articles for eligibility. Any disagreement was subsequently resolved by consensus.

#### Data extraction

From the eligible reports, patient-level data were extracted. Beyond the detailed description of the mutation affecting the *CACNA1C* gene, the following sets of data has been extracted from the source reports (reported in more detail in Supplementary material): (1) Demographic data; (2) Manifestations of the disease (categorized as cardiac or extra-cardiac manifestations); (3) Utilized medical and device therapy; and (4) Outcome of the disease (categorized as death or cardiac events). Major adverse cardiac events (MACE) were defined as death, aborted cardiac arrest (ACA), sudden cardiac death (SCD), or appropriate ICD discharge. Arrhythmia type and circumstances at ACA/SCD/ICD discharge were also recorded if available.

Handling with aggregated data extracted from the original publication by Splawski et al. (10) is detailed in Supplementary material.

### Patient groups

As there was a considerable overlap between genotypes and phenotypes, comparator groups have been defined both based on the genotype and based on the phenotype. Accordingly, patients were divided into the following groups, defined on the genotype:

1) Carriers of exon 8A p.Gly406Arg *CACNA1C* mutations [formerly categorized as Timothy syndrome subtype 1 (TS1)] (4).
2) Carriers of exon 8 p.Gly406Arg *CACNA1C* mutations [formerly categorized as Timothy syndrome subtype 2 (TS2) in the strictest sense] (4).
3) Carriers of exon 8 p.Gly406Arg and pGly402Ser *CACNA1C* mutations [formerly categorized as Timothy syndrome subtype 2 (TS2), according to the original report] (11).
4) Carriers of all exon 8 *CACNA1C* mutations [including mutations p.Gly406Arg, p.Gly402Ser, p.Ser405Arg, p.Gly402Arg, and p.Pro381Ser mutations, formerly categorized as Timothy syndrome subtype 2 (TS2) in the broader sense].
5) Carriers of non-exon 8/8A *CACNA1C* mutations.

According to reported phenotypic manifestations patients were divided into the following three groups:

1) TS (Timothy syndrome, either typical or atypical, defined as patients with characteristic cardiac, and extra-cardiac manifestations). Two subgroups, i.e., typical TS (defined as patients with syndactyly) and atypical TS (defined as patients without syndactyly) were also analyzed separately.
2) Cardiac only TS (COTS, defined as patients with cardiac manifestations only).
3) Isolated long-QT syndrome 8 (LQT8, defined as patients with QTc prolongation only).

Proven mosaic TS cases, reported as such, present in very small numbers in the reported publications, were not considered, taking the very specific genetic constellation of these cases into consideration.

For all the comparator groups two sets of comparisons were made: (i) including index patients only (i.e., one affected person per family) (ii) including all affected patients (i.e., including index patients and all affected family members).

### Re-evaluation of the interpretation of different *CACNA1C* mutations

As many of the considered publications were published before the standards for the interpretation of sequence variants were issued by the American College of Medical Genetics and Genomics and the Association for Molecular Pathology (ACMG/AMP) in 2015, (18) we reassessed the interpretations of all extracted *CACNA1C* mutations. All the mutations were re-evaluated in ClinVar^1^ and Varsome.^2^

### Statistical analysis

For descriptive statistics the number of cases and percentage were calculated for each group in case of categorical and median and interquartile range in case of continuous variables. The differences between groups in case of. categorical variables were examined using the Chi-square test and Fisher’s exact test. For continuous outcomes, because of the origin of the data, non-parametric methods, such as Mann–Whitney *U*-test or the Kruskal–Wallis test was used to detect differences between groups. A *p* < 0.05 value was considered as statistically significant. All calculation was made by IBM SPSS statistical software (IBM SPSS Statistics for Windows, Version 26.0. Armonk, NY, USA: IBM Corp.).

## Results

### Study and patient characteristics

**FIGURE 2:**
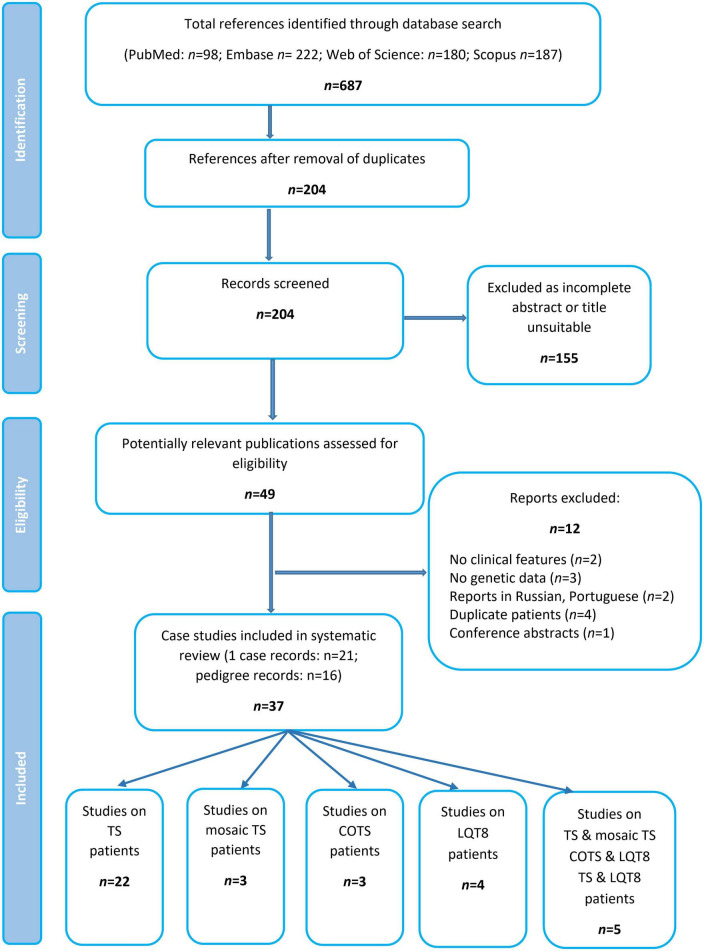
Flow chart of study selection.

Excluding reports on mosaic patients, (19–23) a total of 34 publications comprising data of 134 patients were identified and formed the basis of this systematic review (Figure 2 and Table 1). Most of the publications reported data of one case, whereas 16 studies summarized genotypic and clinical data of more patients/pedigrees.

**TABLE 1:** Main characteristics of the reports included in the study.

References	Year of report	Origin of report	Reported number of index patients	Reported disease phenotype	Reported *CACNA1C*_mutation
Splawski et al. (10)	2004	United States	13	TS	Gly406Arg
Lo-A-Njoe et al. (38)	2005	Netherlands	2	TS	Gly406Arg
Splawski et al. (11)	2005	United States	2	TS	Gly406Arg, Gly402Ser
Jacobs et al. (39)	2006	United States	1	TS	Gly402Ser
Krause et al. (25)	2011	Germany	1	TS	Gly406Arg
Gillis et al. (13)	2012	Canada	1	TS	Ala1473Gly
An et al. (40)	2013	South Korea	1	TS	Gly406Arg
Boczek et al. (8)	2013	United States	4	LQT8	Pro857Arg, Lys834Glu, Pro857Leu, Arg1906Gln
Gao et al. (23)	2013	China	1	TS	Gly406Arg
Fröhler et al. (22)	2014	Lebanon	1	TS	Gly402Ser
Fukuyama et al. (7)	2014	Japan	7	LQT8	P381S, M456I, A582D, R858H, G1783C
Hennessey et al. (15)	2014	Philippines	1	TS	G1911R
Boczek et al. (14)	2015	United States	4	COTS: 3, TS: 1	Arg518Cys, Arg518His, Ile1166Thr
Corona-Rivera et al. (41)	2015	Mexico	1	TS	Gly406Arg
Diep and Seaver (30)	2015	Hawaii	1	TS	Gly406Arg
Ergül et al. (42)	2015	Turkey	1	TS	Gly406Arg
Hiippala et al. (43)	2015	Finland	2	COTS: 1, LQT8: 1	Gly406Arg, Gly402Ser
Wemhöner et al. (6)	2015	Germany	6	TS: 1, LQT8: 5	Ala28Thr, Ile1166Val, Ile1475Met, Arg860Gly, Ile1166Thr, Glu1496Lys
Gunay et al. (44)	2016	Turkey	1	TS	Ala1473Gly
Kawaida et al. (24)	2016	Japan	1	TS	Gly406Arg
Landstrom et al. (26)	2016	Hispanic	1	LQT8	Leu762Phe
Landstrom et al. (26)	2016	Hispanic	1	TS	Gly406Arg
Philipp and Rodriguez (45)	2016	United States	1	TS	Gly406Arg
Sepp et al. (12)	2017	Hungary	1	TS	Gly406Arg
Dufendach et al. (29)	2018	United States	16	TS	Gly402Ser, Gly406Arg, Ser405Arg, Gly402Arg, Lys1211Glu, Cys1021Arg
Gardner et al. (46)	2018	New Zealand	1	LQT8	Arg858His
Kojima et al. (47)	2018	Japan	1	COTS	K1580T
Kosaki et al. (16)	2018	Japan	1	TS	Arg1024Gly
Ozawa et al. (34)	2018	Japan	1	TS	Ser643F
Seo et al. (48)	2018	South Korea	1	COTS	Arg518Cys
Tunca Sahin and Ergul (27)	2018	Turkey	1	TS	Gly406Arg
Walsh et al. (49)	2018	United Kingdom	5	TS	Gly406Arg
Ye et al. (35)	2018	United States	1	LQT8	E1115K
Colson et al. (50)	2019	France	1	TS	Glu407Ala

Out of the 134 patients, there were 85 index patients and 49 additional family members. Out of the 85 index patients 59 suffered from TS, 6 from COTS and 20 from isolated LQT8, respectively. In the entire patient population (index patients and relatives), there were 60 patients with TS, 15 patients with COTS and 59 patients with isolated LQT8 (Table 2).

**TABLE 2:** Patients included into the comparator groups of the study.

	Genotype					Phenotype					Total
		
	*CACNA1C* exon 8A p.Gly406Arg mutation carrier^†‡^	*CACNA1C* exon 8 p.Gly406Arg mutation carrier^†^	*CACNA1C* exon 8 p.Gly406Arg/ p.Gly402Ser mutation carrier^†^	*CACNA1C* exon 8 mutation carrier^†^	*CACNA1C* non-exon 8/8A mutation carrier	Timothy syndrome (TS)			COTS	LQT8	
						
						TS with syndactyly	TS without syndactyly	TS total			
**index patients, *n***	31	6	12	16	33	45	14	59	6	20	85
all patients*, n	31	6	13	17	81	46	14	60	15	59	134

COTS, “cardiac only” Timothy syndrome; LQT8, isolated long QT syndrome, subtype 8. *Including family members. ^†^In 5 patients (all carriers of the p.Gly406Arg mutation) it was not reported whether the patients carried the p.Gly406Arg mutation in exon 8 or in exon 8A. ^‡^Exon 8A mutations carriers all carried the p.Gly406Arg mutation.

### Re-evaluation of the interpretation of different *CACNA1C* mutations

Altogether, 33 *CACNA1C* mutations were extracted from the reports (Table 3). Twenty-eight mutations (85%) had an interpretation of pathogenic (P), likely pathogenic (LP), or variant of unknown significance (VUS) favoring P/LP, either according to ClinVar or Varsome. In general, the number of ClinVar submissions were low (1–4 submissions). Only five mutations (p.Ala28Thr, p.Met456Ile, p.Gly1783Cys, p.Arg1906Gln, p.Gly1911Arg) had a verdict of benign (B), likely benign (LB), or VUS. However, in the case of p.Ala28Thr, reported familial segregation of the mutation in affected family members and functional studies providing evidence for deleterious effect (6) were not considered in the Varsome algorithm which would activate an additional PP1 and a PS3 criteria shifting the interpretation of the variant as VUS favoring LP. Also, for the p.Gly1911Arg variant, reported to be associated with TS, significant differences in the functional analysis and “*de novo”* occurrence of the variant have been demonstrated, (15) activating a PS2 and PS3 criteria and shifting the interpretation of the variant as VUS favoring P. In the case of the p.Met456Ile, Gly1783Cys and Arg1906Gln variants, all reported to be associated with isolated LQT8, no significant additional features (e.g., differences in the functional analysis, familial segregation, etc.) were demonstrated to alter the Varsome verdict. However, for the sake of data integrity all the mutations with the reported phenotypes were considered as they were reported.

**TABLE 3:** Distribution among phenotypes and interpretation of the different *CACNA1C* mutations according to ClinVar and Varsome.

Exon	Codon	Mutation	ClinVar				Varsome		TS (*n* = 59)		COTS (*n* = 6)		LQT8 (*n* = 20)		Total (*n* = 85)	
								
			Verdict	Max	Score	Submiss.	Verdict	Items	*n*	%	*n*	%	*n*	%	*n*	%
2	28	Ala28Thr	VUS	3	3	1	Likely benign	BS2	0	0%	0	0%	1	5%	1	1%
8	381	Pro381Ser	VUS	3	3	1	VUS-favoring LP	PM2, PP3, BP1	0	0%	0	0%	1	5%	1	1%
	402	Gly402Arg	VUS	3	3	1	VUS-favoring P	PM2, PP3, PP5, BP1	1	2%	0	0%	0	0%	1	1%
	402	Gly402Ser	P	5	4.6	3	Likely pathogenic	PP5, PM2, P3, BP1	5	8%	0	0%	1	5%	6	7%
	405	Ser405Arg	ND				Likely pathogenic	PP3, PM2, BP1	2	3%	0	0%	0	0%	2	2%
	406	Gly406Arg	P	5	5	3	Pathogenic	PS1, PP3, PP5, PM2, BP1	6	10%	0	0%	0	0%	6	7%
8A	406	Gly406Arg	P	5	5	3	Pathogenic	PS1, PP3, PP5, PM2, BP1	31	53%	0	0%	0	0%	31	36%
8/8A?	406	Gly406Arg	P	5	5	3	Pathogenic	PS1, PP3, PP5, PM2, BP1	4	7%	1	17%	0	0%	5	6%
9	407	Glu407Ala	ND				VUS-favoring LP	PM2, PP3, BP1	1	2%	0	0%	0	0%	1	1%
	456	Met456Ile	ND				VUS	PM2, BP4	0	0%	0	0%	1	5%	1	1%
12	518	Arg518Cys	P	5	5	2	Likely pathogenic	PP5, PM2, PM5, PP3, BP1	0	0%	3	50%	0	0%	3	4%
	518	Arg518His	P	5	4.7	3	Likely pathogenic	PP5, PM2, PM5, PP3, BP1	0	0%	1	17%	0	0%	1	1%
13	582	Ala582Asp	P	5	5	1	VUS-favoring LP	PM2, PP3, PP5, BP1	0	0%	0	0%	1	5%	1	1%
14	643	Ser643Phe	ND				VUS-favoring LP	PM2, PP3, BP1	1	2%	0	0%	0	0%	1	1%
16	762	Leu762Phe	LP	4	4	1	VUS-favoring LP	PM2, PP3, PP5, BP1	0	0%	0	0%	1	5%	1	1%
18	834	Lys834Glu	P	5	5	1	Likely pathogenic	PS3, Pm2, PP3, BP1	0	0%	0	0%	1	5%	1	1%
19	857	Pro857Arg	P	5	5	1	Likely pathogenic	PS3, PM2, PP3, BP1	0	0%	0	0%	1	5%	1	1%
	857	Pro857Leu	LP	5	4	3	VUS-favoring P	PM2, PM5, PP3, BP1	0	0%	0	0%	1	5%	1	1%
	858	Arg858His	P	5	4.7	4	Likely pathogenic	PP5, PM2, PP3, BP1	0	0%	0	0%	4	20%	4	5%
	860	Arg860Gly	P	5	5	1	VUS-favoring LP	PM2, PP3, PP5, BP1	0	0%	0	0%	1	5%	1	1%
24	1021	Cys1021Arg	VUS	3	3	1	VUS-favoring LP	PM2, PP3, BP1	1	2%	0	0%	0	0%	1	1%
	1024	Arg1024Gly	ND				VUS-favoring LP	PM2, PP3, BP1	1	2%	0	0%	0	0%	1	1%
26	1115	Glu1115Lys	VUS	3	3	1	VUS-favoring LP	PM2, PP3, BP1	0	0%	0	0%	1	5%	1	1%
27	1166	Ile1166Thr	P	5	5	1	VUS-favoring LP	PM2, PP3, PP5, BP1	2	3%	0	0%	0	0%	2	2%
	1166	Ile1166Val	ND				VUS-favoring LP	PM2, PP3, PP5, BP1	0	0%	0	0%	1	5%	1	1%
28	1211	Lys1211Glu	VUS	3	3	1	VUS-favoring LP	PM2, PP3, PP5, BP1	1	2%	0	0%	0	0%	1	1%
36	1475	Ile1475Met	P	5	5	1	VUS-favoring LP	PM2, PP3, PP5, BP1	0	0%	0	0%	1	5%	1	1%
	1496	Glu1496Lys	ND				VUS-favoring LP	PM2, PP3, BP1	0	0%	0	0%	1	5%	1	1%
38	1473	Ala1473Gly	LP	5	4.5	2	VUS-favoring LP	PM2, PP3, PP5, BP1	2	3%	0	0%	0	0%	2	2%
39	1580	Lys1580Thr	ND				VUS-favoring LP	PM2, PP3, BP1	0	0%	1	17%	0	0%	1	1%
42	1783	Gly1783Cys	VUS	3	3	1	Likely benign	PM2, BP1, BP4	0	0%	0	0%	1	5%	1	1%
45	1906	Arg1906Gln	VUS	3	3	8	Benign	BS1, BS2, PP3	0	0%	0	0%	1	5%	1	1%
	1911	Gly1911Arg	VUS	3	3	3	Benign	BS1, BS2	1	2%	0	0%	0	0%	1	1%

A score for all the ClinVar entries were assigned [benign (B): 1, likely benign (LB): 2, variant of unknown significance (VUS): 3, likely pathogenic (LP): 4, pathogenic (P): 5], and the average of the scores were taken into consideration. A score of >4.5 indicated P variants, and a score between 3.5 and 4.5 indicated LP and between 2.5 and 3.5 indicated VUS variants, respectively. ND: no data, TS: Timothy syndrome, COTS: “cardiac only” Timothy syndrome, LQT8: isolated long QT syndrome subtype 8.

### Comparison of patient groups defining different subgroups of Timothy syndrome

#### Comparison of different subgroups of Timothy syndrome, defined on genotype (patients with exon 8A p.Gly406Arg vs. exon 8 p.Gly406Arg mutations; vs. exon 8 p.Gly406Arg/p.Gly402Ser mutations, vs. all exon 8 mutations)

**FIGURE 3:**
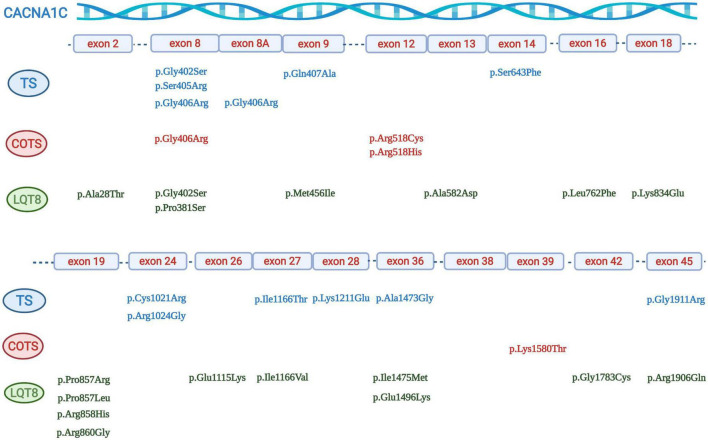
Graphical representation of the location of different *CACNA1C* mutations. Mutations associated with Timothy syndrome are in blue, mutations associated with “cardiac only” Timothy syndrome are in red, and mutations associated with isolated LQT8 are in green.

There were 31 index pts. with exon 8A mutations (all carriers of canonical p.Gly406Arg mutation), 6 pts. with exon 8 p.Gly406Arg mutations, 12 pts. with exon 8 p.Gly406Arg/p.Gly402Ser mutations and 16 pts. with exon 8 mutations (six p.Gly406Arg, six p.Gly402Ser, two p.Ser405Arg, one p.Gly402Arg and one p.Pro381Ser mutations). In further five patients (all carriers of the p.Gly406Arg mutation) it was not unequivocally reported and was impossible to determine whether the patients carried the p.Gly406Arg mutation in exon 8 or in exon 8A. (24–28) (Table 3 and Figure 3).

The detailed comparison of the groups is presented in Table 4. Comparing data on demographics, clinical and ECG manifestations, and outcome, it was only syndactyly which was significantly more frequent in pts. with exon 8A p.Gly406Arg mutations in all comparisons, and baldness which was again more frequent in pts. with exon 8A p.Gly406Arg mutations in comparison with pts. carrying exon 8 p.Gly406Arg/Gly402Ser mutations or all exon 8 mutations. The presence of AV block was also more frequent and the age at MACE was lower in pts. with exon 8A p.Gly406Arg mutations in comparison with pts. with all exon 8 mutations. In addition, patients with exon 8A p.Gly406Arg mutations were significantly younger at the time of diagnosis (median 0 vs. 32 months; *p* = 0.019) and more pts. were diagnosed in the first year of life (89 vs. 44%; *p* = 0.009).

**TABLE 4:** Comparison of clinical characteristics and outcome data in the index patients in Timothy syndrome subtypes.

	*CACNA1C* exon 8A Gly406Arg mutation carrier *n* = 31	*CACNA1C* exon 8 Gly406Arg mutation carrier *n* = 6	*p*	*CACNA1C* exon 8 Gly406Arg/Gly402Ser mutation carrier *n* = 12	*p*	*CACNA1C* all exon 8 mutation carrier *n* = 16	*p*	TS with syndactyly *n* = 45	TS w/o syndactyly *n* = 14	*p*
				
				vs. exon 8A Gly406Arg mutation carrier						
***Demographics***										
Age at diagnosis, months [median (IQR)]	0 (0–11)	0 (0–0)	0.378	5 (0–51)	0.189	**32 (0–63)**	**0.019**	6 (0–24)	0 (0–41)	0.721
Diagnosis at birth, *n* (%)	10/18 (55.6)	5/6 (83.3)	0.351	5/12 (41.7)	0.710	5/16 (31.2)	0.185	14/32 (43.7)	9/14 (64.3)	0.205
Diagnosis in first year of life, n (%)	16/18 (88.9)	5/6 (83.3)	1.000	7/12 (58.3)	0.084	**7/16 (43.7)**	**0.009**	23/32 (71.9)	9/14 (64.3)	0.611
Sex (M/F)	15/9	2/4	0.360	4/6	0.276	4/7	0.156	16/18	8/3	0.143
***Disease manifestation***										
Extra cardiac manifestation, *n* (%)	NA			NA		14/16 (93.3)	0.111	NA		
Syndactyly, n (%)	**29/31 (93.5)**	**1/6 (16.7)**	**0.000**	**4/12 (33.3)**	**0.000**	**6/16 (37.5)**	**0.000**	NA		
Baldness, *n* (%)	18/23 (78.3)	1/2 (50%)	0.430	**1/5 (20)**	**0.026**	**1/8 (12.5)**	**0.002**	**21/27 (77.8)**	**2/7 (20.6)**	**0.024**
Facial abnormality, *n* (%)	7/13 (53.8)	3/6 (50)	1.000	6/12 (50)	1.000	6/16 (37.5)	0.467	14/23 (60.9)	8/14 (57.1)	1.000
Seizures, *n* (%)	3/6 (50)	2/4 (50)	1.000	2/7 (28.6)	0.592	2/8 (25.0)	0.580	5/9 (55.6)	5/9 (55.6)	1.000
Neuro developmental delay, *n* (%)	7/11 (63.6)	5/5 (100)	0.245	8/11 (72.7)	1.000	10/15 (66.7)	1.000	15/21 (63.6)	9/12 (75.0)	1.000
Autism/ASD, *n* (%)	0	0	–	0	–	2/2 (100)	1.000	0/1 (0.0)	1/3 (33.3)	1.000
Recurrent infections, *n* (%)	3/4 (75)	1/2 (50)	1.000	2/5 (40)	0.524	2/6 (33.3)	0.524	4/6 (66.7)	2/4 (50.0)	1.000
Dental abnormalities, *n* (%)	14/15 (93.3)	1/1 (100)	1.000	1/2 (50)	0.228	1/3 (33.3)	0.056	14/14 (100)	3/5 (60.0)	0.058
Hypocalcemia, *n* (%)	0/1 (0.0)	1/1 (100)	1.000	1/1 (100)	1.000	1/2 (100.0)	1.000	1/1 (100)	1/2 (50.0)	1.000
Hypoglycemia, *n* (%)	8/14 (57.1)	2/2 (100)	0.500	2/5 (40)	0.628	2/9 (22.2)	0.197	9/20 (45.0)	4/7 (57.1)	0.678
Orthopedic disorder, *n* (%)	0/1 (0.0)	3/3 (100)	0.250	4/4 (100)	0200	4/5 (80.0)	0.333	3/3 (100)	5/6 (83.3)	1.000
***ECG and arrhythmia manifestations***										
Max. QTc, ms [median (IQR)]	600 (570–650)	666 (555–702)	0.639	610 (555–699)	0.655	603 (555–681)	0.500	603 (570–650)	610 (554–702)	0.991
QTc > 500 ms, *n* (%)	17/18 (94.4)	6/6 (100)	1.000	11/12 (91.7)	1.000	14/16 (87.5)	0.484	30/32 (93.7)	13/14 (92.9)	0.911
AV block, *n* (%)	27/31 (87.1)	6/6 (100)	1.000	7/11 (63.6)	0.174	**7/15 (46.7)**	**0.009**	34/43 (79.1)	9/13 (69.2)	0.472
Syncope, *n* (%)	1/3 (33.3)	2/2 (100)	0.400	4/4 (100)	0.143	4/4 (100.0)	0.143	3/4 (75.0)	4/5 (80.0)	1.000
T wave alternans, *n* (%)	9/14 (64.3)	5/6 (83.3)	0.613	7/12 (58.3)	1.000	9/15 (60.0)	1.000	16/21 (76.2)	6/12 (50.0)	0.149
Documented major arrhythmia NOT leading to ACA/SCD/ICDD, *n* (%)	11/21 (52.4)	4/6 (66.7)	0.662	5/9 (55.6)	1.000	5/9 (55.6)	0.875	13/30 (43.39	7/10 (70.0)	0.169
***Devices and interventions***										
PM, *n* (%)	5/11 (45.5)	4/5 (80.0)	0.308	6/9 (66.7)	0.406	6/9 (66.7)	0.406	9/18 (50.0)	7/10 (70.0)	0.434
ICD/AED, *n* (%)	11/18 (61.1)	3/6 (50.0)	0.665	9/12 (75)	0.694	11/15 (73.3)	0.712	19/29 (65.5)	9/14 (64.3)	1.000
LCSD, *n* (%)	3/18 (16.7)	1/6 (16.7)	1.000	1/12 (8.3)	0.632	1/15 (6.7)	0.607	3/28 (10.7)	2/14 (14.3)	1.000
***Outcome***										
Death, *n* (%)	13/31 (41.9)	2/6 (33.3)	1.000	2/12 (16.7)	0.164	2/16 (12.5)	0.052	16/45 (35.6)	3/14 (21.4)	0.514
Age at death, months [median (IQR)]	3 (1–25)	37 (−)	0.434	37 (−)	0.434	37	0.434	2 (1–18)	44	0.102
Major adverse cardiac event (death/ACA/SCD/ICDD), *n* (%)	22/31 (71.0)	4/6 (66.7)	1.000	10/12 (83.3)	0.698	11/16 (68.7)	0.876	30/44 (68.2)	10/14 (71.4)	1.000
Age at MACE, months [median (IQR)]	24 (2–29)	45 (16–66)	0.151	54 (16–72)	0.062	**54 (16–72)**	**0.034**	**17 (2–30)**	**60 (46–72)**	**0.003**

Statistically different differences are indicated in bold. ASD, autism spectrum disorder; PM, pacemaker; ICD, implantable automated defibrillator; AED, automatic external defibrillator; LCSD, left cervical sympathetic denervation; TS, Timothy syndrome; COTS, “cardiac only” Timothy syndrome; LQT8, isolated long QT syndrome, subtype 8.

Marked QTc prolongation (>500 ms) was present in all the patients, except two patients with exon 8 mutation. The degree of QTc prolongation (maximum QTc) was similar in the groups (median ≥600 ms in all groups). There was no difference in the utilization of pacemaker/ICD implantation or of left cervical sympathectomy. MACE rate was high (67–83%) but was not different in the groups.

#### Comparison of different subgroups of Timothy syndrome, defined on phenotype (Timothy syndromes with or without syndactyly)

The detailed comparison of the groups is presented in Table 4. There were 45 TS pts. with syndactyly and 14 pts. without syndactyly. Only baldness was more frequent and the age at MACE was lower in TS pts. with syndactyly. The degree of QTc prolongation was marked (median ≥600 ms in both groups) and MACE rate was high (68–71%) but showed no statistical difference.

#### Analysis of all patients, including family members

There was one additional family member with exon 8 mutation. His inclusion did not alter any of the statistical comparisons.

### Comparison of patient groups defining different forms of *CACNA1C* gene associated diseases

#### Comparison of different forms of *CACNA1C* gene associated diseases, defined on genotype (patients with exon 8/8A *CACNA1C* mutations vs. non-exon 8/8A *CACNA1C* mutations)

There were 52 index patients with exon 8/8A and 33 index patients with non-exon 8/8A mutations. Exon 8/8A mutations almost all clustered at codon 402 (7 cases), 405 (2 cases), and 406 (42 cases), while the most frequently affected codons in non-exon 8/8A mutation carriers were codon 518 (three cases with p.Arg518Cys mutations and one case with p.Arg518His mutation), codon 857 (one case with p.Pro857Leu and p.Pro857Arg mutations each), codon 858 (four cases with p.Arg858His mutation), codon 1166 (two cases with p.Ile1166Thr, and one case with Ile1166Val mutation) and codon 1473 (two cases with p.Ala1473Gly mutation) (Table 3 and Figure 3).

The detailed comparison of the two groups is presented in Table 5. Patients with exon 8/8A mutations were significantly younger at the time of diagnosis, and a higher percentage of the patients were diagnosed at birth or in the first year of life.

**TABLE 5:** Comparison of clinical characteristics and outcome data in the index patients in *CACNA1C* gene associated disease forms.

	*CACNA1C* exon 8/8A mutation carrier	*CACNA1C* non-exon 8/8A mutation carrier	*p*	TS *n* = 59	COTS *n* = 6	LQT8 *n* = 20	*p*
	*n* = 52	*n* = 33					
***Demographics***							
Age at diagnosis, months [median (IQR)]	**2 (0–30)**	**144 (33–270)**	**<0.001**	**1 (0–30)**	**180 (30–534)**	**174 (144–318)**	**<0.001**
Diagnosis at birth, *n* (%)	**19/39 (48.7)**	**5/31 (16.1)**	**0.005**	**23/46 (50.0)**	**1/4 (25.0)**	**0/20 (0.0)**	**0.000**
Diagnosis in first year of life, *n* (%)	**27/39 (69.2)**	**6/31 (19.4)**	**<0.001**	**32/46 (69.6)**	**1/4 (25.0)**	**0/20 (0.0)**	**<0.001**
Sex (M/F)	19/21	13/18	0.643	24/21	3/3	5/15	0.096
***Disease manifestation***							
Extra cardiac manifestation, *n* (%)	**49/52 (94.2)**	**10/31 (32.3)**	**0.000**				
Syndactyly, *n* (%)	**39/52 (75.0)**	**5/28 (17.9)**	**0.000**				
Baldness, *n* (%)	**20/32 (62.5)**	**3/23 (13.0)**	**0.000**				
Facial abnormality, *n* (%)	15/31 (48.4)	7/27 (25.9)	0.106				
Seizures, *n* (%)	5/14 (35.7)	6/26 (23.1)	0.469				
Neuro developmental delay, *n* (%)	**18/28 (64.3)**	**6/26 (23.1)**	**0.003**				
Autism/ASD, *n* (%)	0/5 (0.0)	2/21 (9.5)	1.000				
Recurrent infections, *n* (%)	**5/10 (50.0)**	**1/21 (4.8)**	**0.007**				
Dental abnormalities, *n* (%)	**15/18 (83.3)**	**2/21 (9.5)**	**0.000**				
Hypocalcemia, *n* (%)	1/3 (33.3)	1/20 (5.0)	0.249				
Hypoglycemia, *n* (%)	**11/24 (45.8)**	**2/25 (8.0)**	**0.004**				
Orthopedic disorder, *n* (%)	**4/6 (66.7)**	**4/23 (17.4)**	**0.033**				
***ECG and arrhythmia manifestations***							
Max. QTc, ms [median (IQR)]	**606 (570–654)**	**498 (475–534)**	**<0.001**	**603 (566–655)**	**490 (480–500)**	**480 (451–498)**	**<0.001**
QTc > 500 ms, *n* (%)	**36/39 (92.3)**	**12/33 (36.4)**	**0.000**	**43/46 (93.5)**	**1/6 (16.7)**	**4/20 (20.0)**	**<0.001**
AVblock, *n* (%)	**38/51 (74.5)**	**6/18 (33.3)**	**0.002**	**43/56 (76.8)**	**1/2 (50.0)**	**0/11 (0.0)**	**<0.001**
Syncope, *n* (%)	5/7 (71.4)	10/13 (76.9)	1.000	7/9 (77.8)	0/2 (0.0)	8/9 (88.9)	0.101
T wave alternans, *n* (%)	20/31 (64.5)	5/9 (55.6)	0.705	22/33 (66.7)	1/2 (50.0)	2/5 (40.0)	0.437
Documented major arrhythmia NOT leading to ACA/SCD/ICDD, *n* (%)	16/34 (47.1)	6/23 (26.1)	0.114	**20/40 (50.0)**	**1/4 (25.0)**	**1/13 (7.7)**	**0.011**
***Devices and interventions***							
PM, *n* (%)	15/24 (62.5)	2/7 (28.6)	0.198	16/28 (57.1)	1/1 (100.0)	0/2 (0.0)	0.196
ICD/AED, *n* (%)	24/37 (64.9)	12/15 (80.0)	0.340	28/43 (65.1)	2/3 (66.7)	6/6 (100.0)	0.214
LCSD, *n* (%)	4/37 (10.8)	3/10 (30.0)	0.155	5/42 (11.9)	0/1 (0.0)	2/4 (50.0)	0.154
***Outcome***							
Death, *n* (%)	**17/52 (32.7)**	**3/33 (9.1)**	**0.017**	**19/59 (32.2)**	**1/6 (16.7)**	**0/20 (0.0)**	**0.006**
Age at death, months [median (IQR)]	2 (1.4–26)	12	0.926	3 (1.1–29)	1.5 (1.5–1.5)		0.560
Major adverse cardiac event (death/ACA/SCD/ICDD), *n* (%)	**37/52 (71.2)**	**11/32 (34.4)**	**0.001**	**40/58 (70.7)**	**2/6 (33.3)**	**6/20 (30.0)**	**0.004**
Age at MACE, months [median (IQR)]	24 (2–53)	60 (20–147)	0.156	27 (2–54)	385 (2–768)	138 (120–156)	0.111

Statistically different differences are indicated in bold. ASD, autism spectrum disorder; PM, pacemaker; ICD, implantable automated defibrillator; AED, automatic external defibrillator; LCSD, left cervical sympathetic denervation; TS, Timothy syndrome; COTS, “cardiac only” Timothy syndrome; LQT8, isolated long QT syndrome, subtype 8.

The predominant phenotype associated with exon 8/8A mutations was TS in 49 patients (96%), COTS in 1 patient (2%) and isolated LQT8 in 2 patient (2%), while with non-exon 8/8A mutations it was TS in 10 patients (29%), COTS in 5 patients (15%) and isolated LQT8 in 18 patients (56%) (*p* < 0.001). Extra-cardiac manifestations (94 vs. 32%; *p* < 0.001) were significantly more frequent in patients with exon 8/8A mutations. As TS was the overwhelmingly prevalent phenotype in patients with exon 8/8A mutations, the major phenotypic characteristics of TS were all significantly more frequent in patients with exon 8/8A mutations. QTc prolongation was present in all the 52 patients with exon 8/8A mutations, while it was seen in only in 79% of the patients with non-exon 8/8A mutations (*p* = 0.0025). The degree of QTc prolongation (maximum QTc) was much more pronounced in patients with exon 8/8A mutations (median 606 vs. 498 ms.; *p* < 0.0001) and the rate of pts. with >500 ms QTc prolongation was much higher (92 vs. 36%; *p* < 0.001). AV block was also observed in significantly more cases in patients with exon 8/8A mutations (74 vs. 33%; *p* = 0.002). There was no difference in the utilization of pacemaker/ICD implantation or of left cervical sympathectomy. There was a marked difference in terms of outcome, as much higher number of pts. with exon 8/8A mutations died (33 vs, 9%; *p* = 0.017) or experienced MACE (71 vs. 34%; *p* = 0.001).

#### Comparison of different forms of *CACNA1C* gene associated diseases, defined on phenotype (patients with Timothy syndromes vs. “cardiac only” Timothy syndrome vs. isolated LQT8)

Out of the 85 index patients there were 59 (69%) with TS, 6 (7%) with COTS and 20 (24%) with the isolated LQT8 phenotype.

As detailed previously, exon 8 or 8A mutations, affecting codons 402, 405 and 406 made up the majority of the genotypes in patients with TS (49/59 patients, 83%). Most mutations leading to COTS affected codon 518 in 4/6 patients (67%). Mutations causing isolated LQT8 scattered through the gene with only one codon having affected in more than one patient (codon 858 in 4/20 patients, 20%) (Table 3 and Figure 3).

The detailed comparison of the three groups is presented in Table 5. Patients with TS were significantly much younger at the time of diagnosis than patients with COTS or isolated LQT8 (median 1 month vs. 180 months vs. 174 months, respectively; *p* < 0.001). In addition, significantly much more pts. with TS were diagnosed at birth or in the first year of life. The degree of QTc prolongation was much more prominent in patients with TS than in patients with COTS or with isolated LQT8 (median 603 vs. 490 vs. 480 ms, respectively; *p* < 0.001) and the number of pts. with >500 ms QTc prolongation was much higher (94 vs. 17% vs. 20%, respectively; *p* < 0.001). There was no significant difference regarding PM/ICD/AED implantation or utilization of left cervical sympathectomy among the groups. There was a marked difference in terms of outcome, as a much higher number of pts. with TS died, as compared with COTS, or isolated LQT8 (32 vs. 17% vs. 0%, respectively; *p* = 0.006) or experienced MACE (71 vs. 33% vs. 30%, respectively; *p* = 0.004).

#### Analysis of all patients, including family members

There was one additional family member with exon 8/8A mutations (total of 53 patients) and additional 48 family members with non-exon 8/8A mutations (total of 81 patients), and there was one additional family member with TS (total of 60 cases), 9 family members with COTS (total of 15 cases) and 39 family members with isolated LQT8 (total of 59 cases).

As family members in every group usually exhibited a milder phenotype, all the statistical differences, observed between the index patient groups regarding disease and outcome parameters, remained statistically different, even at a higher significance level. Furthermore, the difference in age at death or at the time of MACE became statistically different, as patient with exon 8/8A mutations or patients with TS died at an earlier age and had MACE at an earlier age. Data are presented in Supplementary Table 1.

## Discussion

In our systemic review we examined *CACNA1C* gene mutation associated Timothy syndrome, “cardiac only” Timothy syndrome, isolated long QT syndrome 8 and provided data that these disease forms exhibit major differences regarding clinical manifestations and outcome. These differences can be defined either based on the genotype or the phenotype.

In the literature, there is a high degree of controversy regarding the classification of Timothy syndrome. Since the original reports on TS1 and TS2, several proposals have been made to identify TS subtypes. One proposal suggested that all TS phenotypes resulting from mutations in *CACNA1C* should be classified as TS1, and when the next TS disease gene is discovered, it can be classified as TS2 (29). Another proposal recommended that TS1 and TS2 should include exclusively only patients with the p.Gly406Arg mutation in exon 8A (TS1) or in exon 8 (TS2), while the remaining alleles be called atypical TS (4). It has been also argued that there is no clinical utility to distinguishing TS1 from TS2 (30).

However, these proposals were based on small case series and mostly lacked evidence for the most profound question as to whether there is any major difference in the clinical manifestation or, more importantly, the outcome of TS subtypes. Here we provide data, that apart of syndactyly or baldness, there is no major differences regarding clinical manifestations or outcome measures between TS1 and TS2, either defining TS subtypes on the genotype or on the phenotype. Both subtypes are characterized by an extreme degree of QTc prolongation (median ≥600 ms) which is reflected in the similarly high MACE rate. All the above probably makes the distinction between TS1 and TS2 obsolete, at least with regard to clinical outcome, and may make the use of the descriptive terms “classical TS” (with syndactyly) and “non-classical TS” (without syndactyly) more appropriate, as it emphasizes that the two are similar diseases with comparable clinical outcomes.

On the other hand, there are important differences between TS, COTS and isolated LQT8. Based on the phenotype, Timothy syndrome is characterized by a much earlier disease onset, much more pronounced QTc prolongation and much higher mortality than “cardiac only” Timothy syndrome or isolated LQT8. Although phenotypic differences are the basis for the categorization of these disease forms, this is the first time that differences in ECG parameters or in the clinical outcome have been demonstrated. The degree of QTc prolongation is often extreme in TS (>600 ms), and it is >500 ms in >90% of TS patients, while it is only mildly prolonged (<500 ms) in COTS or LQTS. This might be the primary reason for the higher rate of clinical complications, either death or MACE. Similar observations were made by Landstrom et al. based on 28 independent probands (26). Based on the above findings, the established categorization of these disease groups (TS, COTS and isolated LQT8) seems to be justified also on the grounds of clinical outcome.

Differences, like those outlined above, can be encountered if comparisons are made based on the genotype, i.e., between carriers of exon 8/8A mutations vs. non-exon 8/8A mutations. As exon 8/8A mutations are responsible for 83% of TS cases and only 17% of COTS and 10% of isolated LQT8, similar differences can be observed that was seen among TS, COTS and isolated LQT8. These include that disease onset is at much a younger age, phenotypic characteristics of TS are more prevalent, QTc prolongation is much more pronounced and clinical outcome is much more severe in patients with exon 8/8A mutations.

Additionally, the results of our systemic review demonstrated that TS is a rather homogenous disease genetically, as the p.Gly406Arg mutation either in exon 8 or exon 8A alone is responsible for 70% of the cases, and mutations affecting codons 402–407 is responsible for 85% of TS cases. The strongest relationship was seen between mutation p.Gly406Arg in exon 8A and “classical” TS, which is present in 93.5% of the patients. COTS and isolated LQT8 are more heterogenous in this regard having their causative mutations more scattered through the gene.

The cellular electrophysiological alterations caused by the different *CACNA1C* gene mutations alone are not sufficient to explain the above differences in phenotypic expression. *CACNA1C* mutations may result in channel dysfunction in different ways [recently reviewed in detail by Bauer et al. (4)], but the general mechanism is that these mutations lead to gain-of-function alleles of the gene, thereby prolonging the cardiac action potential (AP) and consequently the QT interval (10, 11, 15). The classical mechanism of channel dysfunction, demonstrated for TS mutations (exon 8A and 8 p.Gly406Arg, and exon 8 p.Gly402Ser variants) in different expression systems, is a loss of voltage-dependent inactivation (VDI) of the channel, which leads to prolonged opening of the channel and subsequently causes an increase in the maximum flow of Ca^2+^ through the channel (peak current density) (10, 11, 15). Additional mechanisms, affecting calcium-dependent inactivation (CDI) (31, 32) and steady-state inactivation (SSI) of the channel, significantly increasing the window current, has also been demonstrated (in case of the p.Gly1911Arg mutation) (15). Alternative channel dysfunctional mechanisms are characterized by a reduction in peak current density which is associated with a negative shift in V_1/2_ of activation, the degree of depolarization necessary for activation of the channel (p.Ile1166Thr, p.Arg518His, p.Arg518Cys, p.Ser643Phe, p.Gly419Arg variants) (5, 6, 14, 33–35). This would also result in a net gain-of-function effect on Ca_*v*_1.2 channels.

For explaining the wide variations in phenotypic expression for TS, COTS and isolated LQT8, many different possibilities have been proposed [recently reviewed in detail by Bauer et al. (4)]. One possible mechanism is the presence of parental or individual mosaicism, which has been reported in several cases with TS (20, 22). In the scenario of parental mosaicism, a “*de novo*” mutation may arise during gametogenesis of a parent (who is mosaic for the variant, since only his/her gamete is affected) but the descendant is fully heterozygous for the variant. In the case of individual mosaicism, mosaicism would occur in the developing embryo due to a “*de novo*” mutation which may arise post-zygotically. The variant may be present or absent in the cells and organs of the affected mosaic individual, depending on the timing and location of the mutation happened during embryogenesis (4).

Another explanation could involve the many different isoforms of the *CACNA1C* protein. The transcript profile of the *CACNA1C* gene was reported to be substantially more complex than appreciated by Clark et al., as they identified 38 novel exons and 241 novel transcripts (36). Importantly, many of the novel variants were abundant, and predicted to encode channels with altered function. It has been also demonstrated by Dick et al., based on studies of the p.Gly406Arg mutation in adult guinea pig ventricular myocytes, that cells could tolerate a certain proportion of mutant *CACNA1C* channels (32). A low level of mutant channels caused only a slight AP prolongation; however, above a certain threshold (about 12% for Gly406Arg and about 40% for Gly402Ser), APs became unstable, and cells became arrhythmogenic. This may be further affected by the “repolarization reserve,” as congenital or acquired impairment of one type of transmembrane ion channel does not necessarily result in excessive repolarization changes because other repolarizing currents can take over and compensate (37).

## Conclusion

This current comprehensive systematic review demonstrates that *CACNA1C* gene mutation associated Timothy syndrome, “cardiac only” Timothy syndrome, and isolated long QT syndrome 8 exhibit major differences regarding clinical manifestations and outcome. These differences can be defined either based on the genotype or on the phenotype. Among the phenotypes Timothy syndrome shows the most severe clinical manifestations with much earlier disease onset, much more pronounced QTc prolongation and a much higher mortality than “cardiac only” Timothy syndrome or isolated LQT8. However, distinguishing TS subtypes, in any form, are not supported by our data.

Implications for practice include that a high degree of surveillance is warranted if these diseases, especially TS is identified because of the high rate of adverse cardiac events. Genotyping of the patients help to stratify the risk of the patients as exon 8/8A mutations carry higher risk.

Implications for research include the need for establishing a prospective world-wide registry to fully explore the phenotypic spectrum and clinical outcome of these diseases.

### Limitations

This systematic review harbors all the intrinsic shortcomings of a retrospective study. Given the retrospective nature of the study, clinical information on every aspect of multiorgan involvement in the reported cases was not available. Although all efforts have been done to collect all the cases reported in the literature the study cohorts are still relatively small that limits our ability to draw definitive conclusions. Also, the timespan of the reports encompasses 15 years, from 2004 to 2019, which makes the comparison of individual reports difficult in terms of treatment and outcome over time.

## Data availability statement

The original contributions presented in this study are included in the article/Supplementary material, further inquiries can be directed to the corresponding author.

## Author contributions

All authors listed have made a substantial, direct, intellectual contribution to the work, and approved it for publication.

## Footnotes

1
https://www.ncbi.nlm.nih.gov/clinvar/

2
https://varsome.com/
