# Supplementary material for: Geno- and phenotypic characteristics and clinical outcomes of CACNA1C gene mutation associated Timothy syndrome, “cardiac only” Timothy syndrome and isolated long QT syndrome 8: A systematic review
Source: Front Cardiovasc Med. 2022 Nov 29;9:1021009. doi: 10.3389/fcvm.2022.1021009 (PMC9745330; doi:10.3389/fcvm.2022.1021009)
Supplement: Supplementary file 1 [file Data_Sheet_1.pdf]

Supplementary material:

Methods:

1. Detailed data sets extracted from the reports:

1) Demographic data
    a. age at diagnosis
    b. sex
2) Manifestations of the disease, categorized as
    a. cardiac manifestations (such as structural heart disease, ECG changes including QTc prolongation, bradycardia, fetal/neonatal heart block, documented major arrhythmias, T-wave alternans, syncope, etc.)
    b. extra-cardiac manifestations (such as syndactyly, facial abnormalities, neuro-developmental delay, autism/ARD, baldness, immune disorder, dental abnormalities, hypoglycemia, hypocalcemia, etc.)
3) Utilized medical and device therapy (such as pacemaker and implantable cardioverter defibrillator (ICD) implantation)
4) Outcome of the disease, categorized as
    a. mortality (categorized as disease-related or non-disease-related deaths)
    b. cardiac events [aborted cardiac arrest (ACA), sudden cardiac death (SCD), or appropriate ICD discharge]

2. Handling with aggregated data extracted from the original publication by Splawski et al.

In their original report, Splawski et al. reported 17 patients with Timothy syndrome, in whom 13 genotype positive (*CACNA1C* exon 8A p.Gly406Arg mutation) patients were identified but reported mainly only aggregate data (provided as percentages) on

disease manifestation. To avoid important information loss from one of the largest patient cohorts reported to date we treated data from Splawski's report, as follows:

- regarding disease manifestation, only disease characteristics which were present in >90% of the patients were taken into consideration. These characteristics included QTc prolongation, syndactyly, baldness, dental abnormalities, and congenital heart disease (all present in 100% of the patients) and bradycardia and AV-block (present in 94% of the patients).
- with regard to mortality data, we used the most conservative calculation to define the number of deaths in patients carrying the p.Gly406Arg mutation in Splawski's patient cohort. As 10 patients died in their cohort and there were 4 patients in whom the genotype was not determined, we put the minimum number of deaths in genotype-positive patients as six patients.
