# Supplementary material for: Geno- and phenotypic characteristics and clinical outcomes of CACNA1C gene mutation associated Timothy syndrome, “cardiac only” Timothy syndrome and isolated long QT syndrome 8: A systematic review
Source: Front Cardiovasc Med. 2022 Nov 29;9:1021009. doi: 10.3389/fcvm.2022.1021009 (PMC9745330; doi:10.3389/fcvm.2022.1021009)
Supplement: Supplementary file 2 [file Table_1.pdf]

Supplementary Table 1. Comparison of the clinical characteristics and outcome in all patients (index cases and family members). Statistically different differences are indicated in bold. ASD: autism spectrum disorder; PM: pacemaker; ICD: implantable automated defibrillator; AED: automatic external defibrillator; LCSD: left cervical sympathetic denervation; TS: Timothy syndrome; COTS: 'cardiac only' Timothy syndrome, LQT8: isolated long QT syndrome, subtype 8

	**CACNA1C exon 8/8A mutation carrier** n=53	**CACNA1C non-exon 8/8A mutation carrier** n=81	p	**TS** n=60	**COTS** n=15	**LQT8** n=59	p
Demographics							
Age at diagnosis [months, median (IQR)]	**4 (0-32)**	**168 (36-333)**	**<0,001**	**2 (0-30)**	**294 (60-432)**	**180 (144-414)**	**<0,001**
Diagnosis at birth, n (%)	**19/40 (47,5)**	**5/37 (13,5)**	**0,001**	**23/47 (48,9)**	**1/6 (16,7)**	**0/24 (0,0)**	**0,000**
Diagnosis in first year of life, n (%)	**27/40 (67,5)**	**6/37 (16,2)**	**<0,001**	**32/47 (68,1)**	**1/6 (16,7)**	**0/24 (0,0)**	**<0.001**
Sex (M/F)	20/21	32/47	0,388	25/21	8/7	19/40	0,052
Disease manifestation							
extra cardiac manifestation, n (%)	**50/53 (94,3)**	**10/52 (19,2)**	**<0,001**				
Syndactyly, n (%)	**40/53 (75,5)**	**5/46 (10,9)**	**<0,001**				
Baldness, n (%)	**20/32 (62,5)**	**3/41 (7,3)**	**0,000**				
Facial abnormaility, n (%)	**15/32 (46,9)**	**7/45 (15,6)**	**0,004**				
Seizures, n (%)	5/14 (35,7)	6/44 (13,6)	0,112				
Neuro developmental delay, n (%)	**18/29 (62,1)**	**6/44 (13,6)**	**0,000**				
Autism/ASD, n (%)	0/5 (0,0)	2/39 (5,1)	1,000				
Recurrent infections, n (%)	**5/11 (45,5)**	**1/39 (2,6)**	**0,001**				
Dental abnormailities, n (%)	**15/18 (83,3)**	**2/39 (5,1)**	**0,000**				
Hypocalcaemia, n (%)	1/3 (33,3)	1/38 (2,6)	0,143				
Hypoglycemia, n (%)	**11/24 (45,8)**	**2/43 (4,7)**	**0,000**				
Orthopedic disorder, n (%)	**4/6 (66,7)**	**4/41 (9,8)**	**0,005**				
ECG and arrhythmia manifestations							
Max. QTc [ms, median (IQR)]	**606 (570-652)**	**480 (452-501)**	**<0,001**	**606 (567-654)**	**491 (480-500)**	**476 (450-489)**	**<0,001**
QTc >500 ms, n (%)	**38/40 (95,0)**	**20/69 (29,0)**	**<0,001**	**46/47 (97,9)**	**4/10 (40,0)**	**8/52 (15,4)**	**<0,001**
AV block, n (%)	**38/51 (74,5)**	**6/18 (33,3)**	**0,002**	**43/56 (76,8)**	**1/2 (50,0)**	**0/11 (0,0)**	**<0,001**
Syncope, n (%)	5/7 (71,4)	24/27 (88,9)	0,268	**7/9 (77,8)**	**1/3 (33,3)**	**21/22 (95,5)**	**0,013**
T wave alterns, n (%)	21/32 (65,6)	5/9 (55,6)	0,701	23/34 (67,6)	1/2 (50,0)	2/5 (40,0)	0,425
Documented major arrhythmia NOT leading to ACA/SCD/ICDD, n (%)	**16/35 (45,7)**	**6/66 (9,1)**	**<0,001**	**20/41 (48,8)**	**1/12 (8,3)**	**1/48 (2,1)**	**<0,001**
Devices and interventions							
PM, n (%)	15/25 (60,0)	3/8 (37,5)	0,418	16/29 (55,2)	2/2 (100,0)	0/2 (0,0)	0,164
ICD/AED, n (%)	25/38 (65,8)	14/22 (63,6)	1,000	29/44 (65,9)	3/4 (75,0)	7/12 (58,3)	0,897
LCSD, n (%)	5/38 (13,2)	3/10 (30,0)	0,336	6/43 (14,0)	0/1 (0,0)	2/4 (50,0)	0,274
Outcome							
Death, n (%)	**17/53 (32,1)**	**8/81 (9,9)**	**0,003**	**19/60 (35,0)**	**4/15 (26,7)**	**2/59 (3,4)**	**0,000**
Age at death [months, median (IQR)]	**2 (1,4-25,5)**	**300 (28-396)**	**0,027**	**3 (1-29)**	**324 (145-396)**	**372 (312-432)**	**0,029**
Major adverse cardiac event (death/ACA/SCD/ICDD), n (%)	**38/53 (71,7)**	**18/80 (22,5)**	**0,000**	**41/59 (69,5)**	**5/15 (33,3)**	**10/59 (16,9)**	**<0,001**
Age at MACE [months, median (IQR)]	**27 (2-51)**	**222 (52-396)**	**0,003**	**30 (2-53)**	**360 (216-516)**	**234 (138-372)**	**0,003**
